# Effects of the Administration of a Non-specific Immune Stimulant Around Transportation on Health and Performance of Jersey and Jersey-Cross Heifer Calves During the Rearing Period: Randomized Clinical Trial

**DOI:** 10.3389/fvets.2020.550202

**Published:** 2020-10-14

**Authors:** Bobwealth O. Omontese, Luciano S. Caixeta, Vinicius S. Machado, Aaron Rendahl, Maria L. K. Celestino, Paulo R. Menta, Daniela Paiva, Angel Garcia-Muñoz, Aleksandar Masic

**Affiliations:** ^1^Department of Veterinary Population Sciences, College of Veterinary Medicine, University of Minnesota, Saint Paul, MN, United States; ^2^Department of Food and Animal Sciences, College of Agricultural, Life and Natural Sciences, Alabama A and M University, Huntsville, AL, United States; ^3^Department of Veterinary Sciences, College of Agricultural Sciences and Natural Resources, Texas Tech University, Lubbock, TX, United States; ^4^Department of Veterinary and Biomedical Sciences, College of Veterinary Medicine, University of Minnesota, Saint Paul, MN, United States; ^5^Faculty of Veterinary Sciences, University Cardenal Herrera CEU, CEU Universities, Valencia, Spain; ^6^NovaVive Inc., Napanee, ON, Canada

**Keywords:** Jersey calves, mortality, immune stimulant, disease treatment, average daily gain (ADG)

## Abstract

Our objective was to evaluate the effects of a non-specific immune stimulant (IS) administered around transportation on health scores (HS), average daily gain (ADG), disease treatment and mortality of Jersey and Jersey-cross calves during the rearing period. Newborn calves (4 d ± 1) were randomly allocated to receive either 1 mL of saline (CON; *n* = 438), 1 mL of IS before transport (BTIS; *n* = 431), or 1 mL of IS immediately after transport (ATIS; *n* = 436). Calves were health scored weekly for 3 weeks after transport. The data were analyzed using multivariable linear mixed models and multivariable logistic regression models. Kaplan-Meier survival analysis was performed for time to event analysis. Treatment, birth weight, breed, site of birth, serum total solids, dam parity, season of enrollment, and metaphylaxis were offered to models. Differences in respiratory and fecal HS, and ADG between treatment groups were not statistically significant. A total of 196 (15.0%) calves were treated at least once for any disease and 52 calves were treated multiple times. The proportion of calves treated for respiratory disease and/or diarrhea were 14.4, 14.4, and 16.2% for BTIS, ATIS and CON groups, respectively. Although the differences in the likelihood of treatment for both respiratory disease and/or diarrhea during the first 9 weeks of life was not statistically different between groups, we observed that more calves in the control group received disease treatments around 15 days of age compared with calves that received IS. The likelihood of treatment for respiratory diseases alone during the first 30 days of life was smaller in the calves that received IS before transportation when compared to the control group. Only 18 (1.4%) calves died within the study period. The calf mortality likelihood was not statistically different between study groups; however, fewer calves in the IS groups died when compared to CON. In conclusion, the use of IS around transportation did not influence weekly HS, ADG, and the number of disease treatments during the rearing period, but administering IS before transportation resulted in fewer treatments of respiratory diseases during the first 30 days post-transport and marginally lower mortality rates during the rearing period.

## Introduction

The occurrence of diseases during the rearing period in dairy heifers is associated with impaired productivity of dairy cows during first lactation (1–4). Thus, calf-hood well-being is important to the economic success of dairy operations. Among the morbidities affecting dairy calves during the rearing period, respiratory diseases (i.e., pneumonia) and diarrhea have been reported as the most prevalent and economically important (3, 5–7). In the United States, ~11 and 19% of calves show signs of pneumonia and diarrhea at least once, respectively, and 5% die before weaning (7). One of the reasons for the high morbidity among pre-weaned calves is the fact that dairy calves are born nearly agammaglobulinemic and are extremely dependent on acquisition of maternal immune protection through proper ingestion of colostrum immediately after birth (8, 9). Successful passive transfer of maternal immunoglobulins is important to assist with protection against infectious agents by providing specific antibodies and to enhance the cell-mediated immune response in calves (10–13). Although extensive research has demonstrated the importance of adequate passive transfer of immunity to calf health, the quality and quantity of colostrum offered to newborn calves are often inadequate. Consequently, dairy calves are susceptible to infectious diseases early in life and antibiotics are often used to treat pneumonia and diarrhea in commercial dairy farms. According to a nationwide survey in the United States, ~25% of calves receive an antibiotic for the treatment of illness during the pre-weaning period (7).

Poor housing, inadequate ventilation and transportation are some of the stressful conditions associated with high disease incidence in pre-weaned dairy calves (8). A review by Van Engen and Coetzee (14) described the intricate role of transportation on immune suppression and increased inflammation, pre-disposing feedlot cattle to pneumonia. Transportation increases disease susceptibility of calves (15) and performing preventive interventions before transportation is associated with enhanced health and performance after transportation (16). However, vaccinations and metaphylaxis are often performed after transportation (17). Treating dairy calves after the disease is diagnosed does not eliminate the negative effects on long-term production and the metaphylactic use of antimicrobials can contribute to the alleged influence of animal agriculture on the selection of antimicrobial resistance genes (18, 19). Thus, there is a need to investigate alternative strategies that can enhance animal health around transportation without the use of antibiotics.

Immune stimulants (IS) offer an alternative method to activate innate immune response of newborn dairy calves and IS have the potential to decrease antibiotic treatments for pneumonia and diarrhea in calf operations during the rearing period (20, 21). Among the products available on the market, a mycobacterium cell wall fraction immune stimulant is approved for the reduction of clinical signs and mortality associated with K99 *Escherichia coli* diarrhea in neonatal calves (Amplimune®, NovaVive Inc., Napanee, ON, Canada). Additionally, mycobacterium cell wall fraction immune stimulants have been shown to modulate innate immune response and stimulate lymphocyte functional activity, *in vivo* and *in vitro*, in other species within hours of administration and last only for a few days (22–25). Considering that immune stimulation can induce early activation of the non-specific innate immune system of newborn dairy calves and provide the first line of defense against microbial pathogens (26, 27), this study was designed to evaluate the effects of this commercially available IS on health and performance of pre-weaned Jersey and Jersey-cross calves following transportation. Our hypothesis was that the use of the IS would improve health and performance due to an improved immune response in newborn calves around transportation, leading to improved health and performance during the pre-weaning period. Our specific objectives were to determine whether this non-specific immune stimulant would improve weekly health score (HS) during the first 3 weeks post-treatment, improve average daily gain (ADG) and decrease disease treatments and mortality of calves transported within their first week of life during the rearing period.

## Materials and Methods

All experimental protocols were approved by the Institutional Animal Care and Use Committee (IACUC) of the University of Minnesota and Texas Tech University.

### Study Design, Calves Management and Data Collection

This randomized controlled clinical trial was conducted in a commercial dairy system from March to December 2018. Calves were born at nine different sites from the same dairy system in Minnesota, immediately separated from their dam after birth, weighed, fed colostrum (4 L within 6 h after birth), and transported to the initial temporary holding facility where they were housed for 3–4 days (depending on day of birth) before transportation. Management and standard operating procedures were the same in the nine different origin sites. In the study facility, calves were placed in individual hutches bedded with straw inside a large cross-ventilated barn.

Newborn Jersey and Jersey-cross heifer calves were enrolled between 3 and 5 days of life. Only multiparous dairy cows were present in the Minnesota sites of this particular dairy system, therefore no calves born from primiparous animals were enrolled in this study. A day prior to enrollment, the list of eligible calves were allocated randomly to treatments using the Microsoft Excel 2016 randomization generator (Microsoft Corporation, Redmond, WA) by the corresponding author. At the temporary holding facility, calves received 1.8 L per feeding of a reconstitute milk replacer (27% crude protein, 25% crude fat, DM basis) two times a day, *ad libitum* water in individual feeding bottles, and were checked for general health. Briefly, sick calves were identified based on whether they consumed the entire milk replacer volume offered, signs of weakness (i.e., unable to rise), diarrhea or any other visible abnormalities. Sick calves were treated by the on-farm veterinarian according to farm protocols and transportation to the heifer growing facility in New Mexico was withheld until the illness resolved. For this reasons, visibly sick calves were not enrolled in the study. Calves were randomly allocated to receive one of three treatments:(1) 1 mL of sterilized saline (CON); (2) 1 mL of IS before transport to grower facility (BTIS); or (3) 1 mL of IS on arrival (after transportation) at the grower facility (ATIS). All treatments were administered subcutaneously on the neck and within 2 h before (CON and BTIS) or after (ATIS) transportation by the University of Minnesota (BTIS) and Texas Tech University (ATIS) research teams, respectively. All calves were safely loaded into a truck and transported to the calf-rearing facility (~18 h of transport). In order to facilitate identification of study calves requiring treatment at arrival at the growing facility, calves were fitted with removable plastic ear clips. The ear clips were removed immediately after administration of IS to the calves in the ATIS group to ensure that the assigned treatment stayed masked for the research personnel performing health scoring of animals during the first 3 weeks post-transport at the grower facility, and that farm personnel were also masked when identifying and treating sick animals.

At enrollment, blood samples were collected by jugular venipuncture using Vacutainer tubes (10 mL BD Vacutainer glass serum tubes; Becton Dickinson, Franklin Lakes, NJ) from all calves for the determination of serum total solids concentration. Samples were placed immediately in ice and later centrifuged at 2,000 × *g* for 15 min at 4°C for serum separation. Serum total solids were measured using a digital refractometer (MISCO; Palm Abbe PA203X, Whitewater, WI) to evaluate colostrum management of the farm and failure of passive transfer was defined as serum total solids <5.5 g/dL (28).

At the calf-rearing facility, heifer calves were housed in individual hutches bedded with straw, received on average 1.8 L per feeding of a reconstituted milk replacer (27% crude protein, 22% crude fat, DM basis) two times a day, and had *ad-libitum* access to water and calf starter throughout the rearing period. Calf health was evaluated weekly during the first 3 weeks post-transport using a modified calf health scoring system adapted from McGuirk and Peek (29). Briefly, individual health score measures rectal temperature, cough, nasal discharge, ocular discharge (eye score), ear position (ear score), and fecal consistency were scored from 0 to 3. For all categories, lower scores for individual health measures indicated apparently healthier animals. Health score was assessed by one trained observer from the Texas Tech University research team during a weekly visit to the heifer raising facility during the 3 weeks following transportation. For the purpose of this study, a veterinary attention score was created based on respiratory and fecal scores for each week post-transport separately. Calves with respiratory score >4 and calves with fecal score >2 were considered as in need of veterinary attention because of respiratory disease and diarrhea, respectively. Although the research group assessed HS in study animals on a weekly basis during the first 3 weeks post-transport, HS results and veterinarian attention recommendation was not made available for farm personnel in order to avoid deviations from farm standard operating procedures. The HS data was collected and used as an objective measurement of health status post-transportation, however, only animals that received treatments by farm personnel were considered sick for disease treatment analysis.

According to farm protocols, calves were considered sick when clinical signs including weakness, depression, rectal temperatures of over 40°C (>104°F), difficult, shallow or rapid breathing, dehydration, nasal discharge, diminished appetite, coughing, or watery stools were observed. Treatments followed farm protocols and standard operating procedures and were developed by the on-farm veterinarian. Treatment information including treatment number, date, and farm diagnosis was recorded on on-farm management software (Dairy Comp 305; Valley Ag Software, Tulare, CA). Disease treatment records for the first 9 weeks of life (63 days of age) were used for statistical analysis. Additionally, beginning in September 2018 farm management implemented a metaphylactic treatment (Zuprevo, Tildipirosin, 4 mg/kg of body weight; Merck Animal Health, Summit, NJ) to all calves (*n* = 457) at the facility at 35 days of life. The implementation of the metaphylactic treatment was unrelated to our study and had the goal to decrease a perceived higher occurrence of respiratory cases around 40 days of life. This perceived higher occurrence of respiratory cases was not a disease outbreak. The metaphylactic treatment did not fulfill farm management expectations and was halted few months after the end of our study. Animals in all treatment groups received the metaphylactic treatment and hence a metaphylactic treatment variable was added to the statistical models. All heifer calves were weighed using a portable digital scale (Raytec® 42′ Calf Cart™, Raytec LLC, Ephrata, PA) at 9 weeks of age (~ 63 days of life).

### Statistical Analyses

Sample size was calculated using JMP 14 (SAS Inst., Cary, NC). Sample size calculation was performed based on previous reports of the disease incidence (i.e., pneumonia and diarrhea) in the United States dairy calves and the farm's historical data within the rearing period. We were expecting to see a reduction in disease treatment from 40 to 30% following treatment with IS. Therefore, a minimum of 294 calves per treatment was required to detect a reduction of 10 percentage points in the incidence of calf-hood diseases treatments between control and IS treatment groups, with 80% power at a 5% significance level. Prior to the beginning of the study, we inflated our sample size by 20% to account to loss of follow up (~60 animals per group). After preliminary descriptive statistical analysis when the expected number of animals completed the trial and the lower disease treatment rates were observed, the research team decided to enroll animals for another 3 weeks (maximum allowed based on budgetary constraints) as an attempt to achieve sufficient numbers to capture the expected differences.

Incidence of calf diseases and mortality are expressed in percentages. The effect of IS treatment on weekly HS were analyzed for each week post-transport individually. Respiratory and fecal scores were analyzed separately as a continuous variable using a generalized linear model and as a dichotomous outcome based on the calculated veterinary attention score by separate chi-squared test. Average daily gain was calculated by dividing the change in weight by the number of days between birth and weaning and was evaluated using multivariable linear regression. Statistical analyses for disease treatment during the rearing period (9 weeks), treatment of respiratory diseases during the first 30 days, mortality, and re-treatment of calves for respiratory disease or diarrhea were carried out using multivariable logistic regression. In addition to treatment, the following independent variables were included in the models to account for their association with each given outcome: season of enrollment (season 1 = March and April, season 2 = May and June, Season 3 = August and September, season 4 = October and November), breed (Jersey or Jersey-cross), birth weight, site of birth, serum total solids, dam parity (lactation = 2; lactation > 3). Metaphylaxis was included as a covariate to the ADG model and to all disease treatment and mortality models that accounted for the entire rearing period. Homoscedasticity and independence of error assumptions was assessed by visual observation of models' residual plots and the Hosmer-Lemeshow test was used to test goodness-of-fit of logistic models.

Kaplan-Meier survival analysis was performed to show the survival of calves from disease treatment or mortality during the rearing period, and the time to respiratory disease treatment during the first 30 days of life. For the time to first disease treatment analysis, calves were right-censored if dead before receiving treatment for any disease or if they did not receive any treatment until the last day of the follow up period when final weights were measured. For the time to respiratory disease treatment event before 30 days, similar strategy for censoring data was applied but follow up period was arbitrarily set to end at 30 days of age. For the time to death analysis, calves were right-censored if they were alive at the end of the data collection period when final weight data was collected (9 weeks of life). Backward stepwise elimination process was used to create the most parsimonious statistical models. Treatment and metaphylaxis (when present) were forced into all statistical models while all other covariates were excluded if *P* > 0.20. Differences with *P* < 0.05 were considered statistically significant. Statistical analyses were performed using SAS version 9.4 (SAS Institute Inc., Cary, NC) and Kaplan-Meier curves were created in R 3.6.0 (30).

## Results

### Descriptive Analysis

A total of 1,332 heifer calves were enrolled in the study; however, 27 calves were excluded from statistical analysis. Reasons for exclusion were lost of follow up (*n* = 14) and development of morbidities (*n* = 13; four calves from CON, four calves from BTIS, and six calves from ATIS) that were not defined prior to the beginning of the study (i.e., arthritis, navel infection, or pink eye). Therefore, 1,305 calves including Jersey (*n* = 568) and Jersey-cross (*n* = 737) completed the study. Information on the number of animals from each breed, dam parity, age at enrollment, birth weight, serum total solids at enrollment, age at weaning, final weight (weaning weight), and number of animals that received metaphylactic treatment are presented in Table 1. No numerical differences were observed between the three treatment groups at enrollment. Furthermore, no adverse reaction after the administration of the IS subcutaneously was observed during study.

**Table 1:** Descriptive characteristics (mean ± SD) of Jersey and Jersey-cross calves enrolled in a study to evaluate the effects of a non-specific immune stimulant on calf health and performance during the rearing period.

**Variable**	**Treatment**		
	**Control (*n* = 438)**	**BTIS^a^ (*n* = 431)**	**ATIS^b^ (*n* = 436)**
**Breed**			
Jersey	200	189	179
Jersey-cross^c^	238	242	257
Dam parity	2.64 ± 0.82	2.60 ± 0.83	2.65 ± 0.88
Age at enrollment, *d*	4.86 ± 0.34	4.83 ± 0.39	4.83 ± 0.39
Birth weight, kg	31.7 ± 4.3	32.0 ± 4.4	31.9 ± 4.5
Serum total solid, g/dL	6.60 ± 0.62	6.66 ± 0.61	6.59 ± 0.62
Age at weaning, d	61.3 ± 1.5	61.2 ± 1.5	61.2 ± 1.4
Weaning weight, kg	59.9 ± 7.9	60.6 ± 8.1	60.3 ± 8.0
Metaphylaxis^d^, *n* (%)	153 (35)	151 (35)	143 (33)

a *BTIS = before transport immune stimulant*.

b *ATIS = after transport immune stimulant*.

c *Jersey and Holstein cross heifer calves*.

d *Subcutaneous administration of Zuprevo (Tildipirosin, 4 mg/kg of body weight; Merck Animal Health, Summit, NJ) at 35 days of age*.

### Weekly Health Scores

Weekly health scores are presented in Table 2. Overall, there were no statistical differences in respiratory score when comparing BTIS and ATIS with CON during the 3 weeks when HS was assessed. A numerical difference was observed for fecal score during week 1 post-transport (*P* = 0.06). We did not observe a difference in the number of calves that required veterinary attention based on total respiratory score (total respiratory score >4) nor fecal score (total fecal score >2) within each week. The percentage of calves that required veterinary attention based on fecal scores decreased throughout the 3-week period post-transport in all treatment groups while a similar percentage of calves were considered to require veterinary attention based on respiratory scores during the same period.

**Table 2:** Proportion of Jersey and Jersey-cross calves with recommended veterinary attention based on health scores^a^ during the first 3 weeks after transportation.

**Week after transport**	**Treatment**			
	**Control (*n* = 438)**	**BTIS^b^ (*n* = 431)**	**ATIS^c^ (*n* = 436)**	***P*-value**
**Week 1**				
Respiratory score^d^, mean ± SD	1.42 ± 0.99	1.37 ± 0.92	1.49 ± 0.99	0.21
Fecal score^e^, mean ± SD	1.12 ± 1.21	1.14 ± 1.21	1.30 ± 1.24	0.06
Respiratory score–Attention^f^, *n* (%)	13 (3%)	10 (2%)	13 (3%)	0.79
Fecal score–Attention^g^, *n* (%)	166 (38%)	162 (38%)	186 (43%)	0.23
**Week 2**				
Respiratory score	1.37 ± 1.02	1.36 ± 1.01	1.39 ± 1.03	0.73
Fecal Score	0.90 ± 1.12	0.93 ± 1.15	0.96 ± 1.15	0.58
Respiratory score–Attention	18 (4%)	18 (4%)	12 (3%)	0.45
Fecal score–Attention	91 (21%)	95 (22%)	76 (17%)	0.21
**Week 3**				
Respiratory score	1.22 ± 1.04	1.16 ± 0.91	1.20 ± 0.99	0.63
Fecal Score	0.47 ± 0.92	0.48 ± 0.90	0.43 ± 0.85	0.71
Respiratory score–Attention	9 (2%)	6 (1%)	11 (2%)	0.48
Fecal score–Attention	61 (14%)	63 (15%)	48 (11%)	0.25

a *Weekly health score was evaluated for all the calves during the first 3 weeks of life using a calf health scoring systems adapted from McGuirk, University of Wisconsin*.

b *BTIS = before transport immune stimulant*.

c *ATIS = after transport immune stimulant*.

d *Respiratory score = Mean respiratory score per treatment group*.

e *Fecal score = Mean fecal score per treatment group*.

f *Respiratory score–Attention = Veterinary attention because of respiratory diseases was defined as positive when total respiratory score was equal or >4 based on the health scoring systems adapted from McGuirk, University of Wisconsin (dichotomous outcome)*.

g *Fecal score–Attention = Veterinary attention because of diarrhea was defined as positive when total fecal score was equal or >2 based on the health scoring systems adapted from McGuirk, University of Wisconsin (dichotomous outcome)*.

### Average Daily Gain

There were no differences in ADG when comparing treatments during the rearing period (*P* = 0.58). Calves in the control group gained an average of 460 g daily (range = 156–699 g/d), while calves in the BTIS group gained an average of 466 g daily (range = 159–796 g/d), and calves in the ATIS group gained 463 g daily (range = −14–729 g/d). Calves born in May and June had a lower ADG (*P* < 0.001) when compared to calves born in March and April, while calves born in October and November had a greater ADG (*P* < 0.001) when compared to the same referent group of calves. ADG gain was greater (*P* < 0.001) in Jersey-cross calves when compared to Jersey calves, greater (*P* < 0.001) in calves that received methaphylactic treatment during the hearing period when compared to calves that did not receive metaphylaxis, and it was associated with birthweight (*P* < 0.001). Lastly, calves born in all but one of the birth sites had similar ADG when compared the referent birth site (Table 3). ADG ranged from 338 to 583 g depending on the week of study when calves were enrollment (week 10 and week 18, respectively).

**Table 3:** Multivariable linear model evaluating the effects of a non-specific immune stimulant around transportation, season of enrollment, birth weight, breed, site of birth, dam parity, and metaphylaxis on average daily gain of Jersey and Jersey-cross calves during the rearing period.

**Variable**	**Estimate**	**Standard error**	***P*-value**
Intercept	0.657	0.05	<0.001
**Treatment**^a^			
Control	Referent		
BTIS	0.005	0.005	0.32
ATIS	0.004	0.005	0.45
**Season**^b^			
March–April	Referent		
May–June	−0.053	0.01	<0.001
August–September	0.009	0.01	0.33
October–November	0.048	0.01	<0.001
Birth weight	−0.001	0.00	<0.001
**Breed**^c^			
Jersey	Referent		
Jersey-bred	0.029	0.005	<0.001
**Source site**			
Birth site A	Referent		
Birth site B	−0.028	0.02	0.27
Birth site C	0.012	0.01	0.49
Birth site D	−0.001	0.01	0.98
Birth site E	−0.026	0.01	0.05
Birth site F	0.009	0.01	0.58
Birth site G	−0.005	0.01	0.77
Birth site H	−0.006	0.01	0.69
Birth site I	0.026	0.01	0.12
**Parity**^d^			
Lactation = 2	Referent		
Lactation > 3	−0.008	0.005	0.10
**Metaphylaxis**^e^			
No	Referent		
Yes	0.09	0.005	<0.001

a *Treatment: Calves received subcutaneous administration of 1 mL of a non-specific immune stimulant at 4 ± 1 days of life. CON = calves that receive saline before transport (n = 438); BTIS = calves that received immune stimulant before transport (n = 431) and ATIS = immune stimulant after transport (n = 436)*.

b *Enrollment season: Period of the study referent to the week when first set of claves was enrolled. Calves were enrolled on a weekly basis from March to November of 2018*.

c *Breed: Jersey (n = 568) and Jersey-Holstein cross (n = 737) heifer calves were enrolled in the study*.

d *Dam parity: Dam parity was dichotomized (lactation = 2 and lactation > 3) based on the lactation that dams were starting. Only multiparous cows were housed in the different sites where study calves were born*.

e *Starting in September 2018 farm management implemented a metaphylactic treatment (Zuprevo, Tildipirosin, 4 mg/kg of body weight; Merck Animal Health, Summit, NJ)*.

### Disease Treatment and Mortality

A total of 196 (15.0%) calves were treated at least once and 18 (1.4%) calves died during their first 9 weeks of life. The proportion of animals treated for pneumonia and/or diarrhea within each group was 14.4, 14.4, and 16.2% for BTIS, ATIS and CON groups, respectively. Treatments for pneumonia alone accounted for 163 (61.3%) of the 266 treatments administered during the study period, treatments for diarrhea accounted for 93 (35.0%), and treatments for both diseases at the same time accounted for 10 (3.7%). Of the total number of calves treated within each group during the study period, 52 calves were treated multiple times, 13 (3.0%) in the BTIS group, 19 (4.4%) in the ATIS group, and 20 (4.6%) from the CON group. One hundred and eighty-nine (71.1%) of all disease treatments occurred within the first 30 days of life. The cumulative incidence of disease treatments and mortality per treatment group during the rearing period by treatment groups is presented in Table 4.

**Table 4:** Cumulative incidence of disease treatments and mortality during the rearing period for newborn Jersey and Jersey-cross calves receiving subcutaneous administration of a non-specific immune stimulant around transportation during the rearing period (9 weeks).

**Variable**	**Treatment**				
	**Control (*n* = 438)**	**BTIS^a^ (*n* = 431)**	**ATIS^b^ (*n* = 436)**	***P*-value^c^**	**Contrast^d^**
Disease treatment, *n* (%)					
Pneumonia	55 (12.5)	49 (11.3)	62 (14.2)	0.66	0.60
Diarrhea	38 (8.7)	29 (6.7)	26 (6.0)	0.81	0.68
Pneumonia and diarrhea	4 (0.9)	3 (0.7)	3 (0.7)	0.71	0.49
Mortality, *n* (%)	10 (2.3)	4 (0.9)	4 (0.9)	0.16	0.05

a *BTIS = before transport immune stimulant*.

b *ATIS = after transport immune stimulant*.

c *Overall P-value*.

d *P-value when comparing both treatment groups combined vs. the control group*.

**Figure 1:**
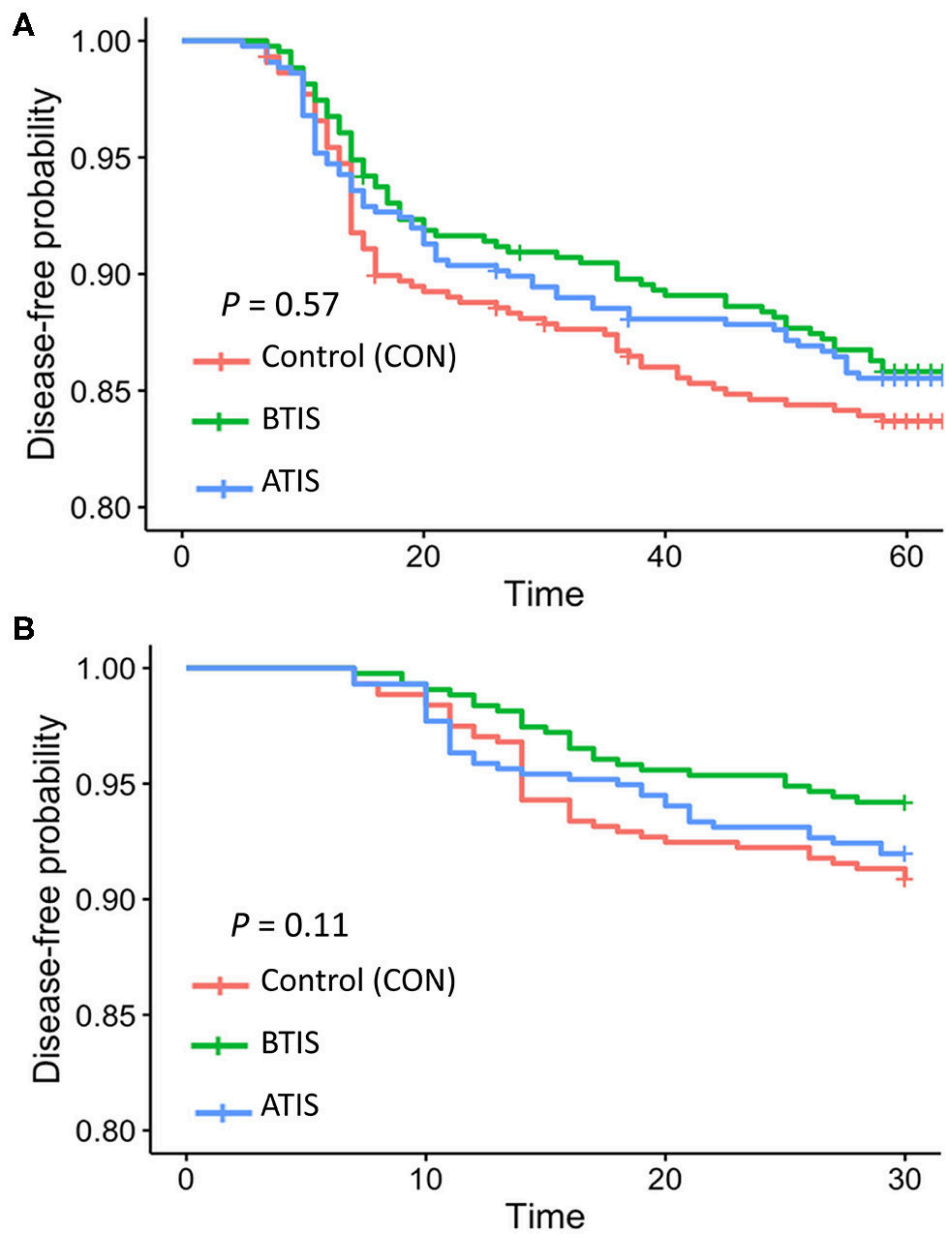
Kaplan-Meier survival curves showing the cumulative proportion of calves that received treatment for any disease during the first 9 weeks of life **(A)** and calves that received treatment for respiratory diseases during the first 30 days of life **(B)**. CON = calves that receive saline before transport (*n* = 438; red line); BTIS = calves that received immune stimulant before transport (*n* = 431; green line) and ATIS = immune stimulant after transport (*n* = 436; blue line).

Multivariable logistic regression models were developed to determine the odds of receiving a disease treatment during the rearing period. No differences in the odds of receiving a disease treatment during the entire rearing period was observed when comparing all experimental groups (Table 5). However, we observed that more calves in the control group received treatment for pneumonia and/or diarrhea around 15 days of age compared with calves that received IS (Figure 1A). The observed change in the Kaplan-Meier curve from day 11 to day 20 was 8.5 percentage points for control, compared with 6.3 and 5.5, for BTIS and ATIS, respectively, and the overall estimated proportion receiving treatment by day 30 was 12.2% for CON, compared with 9.1 and 10.5% for BTIS and ATIS, respectively. Season of enrollment was associated (*P* = 0.02) with different likelihood of receiving a disease treatment during the rearing period while birth weight (*P* = 0.05) and breed (*P* = 0.08) were only marginally associated with the odds of receiving a disease treatment during the same period. Calves born in May and June were more likely to have a treatment event during the rearing period than calves born in March and April (OR = 1.05; 95% CI: 0.69–1.60; *P* = 0.02) while calves born in October and November had a lower likelihood of receiving disease treatment during the rearing period (OR = 0.40; 95% CI: 0.22–0.74; *P* = 0.003). The odds of receiving a treatment during the rearing period was smaller for Jersey-cross calves when compared to Jersey calves (OR = 0.73; 95% CI: 0.52–1.04; *P* = 0.05), and the likelihood of receiving a treatment for any disease during the rearing period was smaller in calves that were heavier at birth (OR = 0.98; 95% CI: 0.96–0.99; *P* = 0.05). When controlling for all other variables, calves that received metaphylactic treatment at 35 d of age had a 40% lower likelihood of receiving treatment for a disease during the rearing period (OR = 0.60; 95% CI: 0.37–0.97; *P* = 0.04) when compared to calves that did not receive metaphylactic treatment (Table 5). When analyzing treatments for respiratory disease alone during the first 30 days of life, we found that BTIS had a significantly lower odds of receiving a treatment for respiratory disease compared with CON (OR = 0.53; 95% CI: 0.29–0.97; *P* = 0.03). No difference was observed when comparing ATIS (OR = 0.92; 95% CI: 0.54–1.55; *P* = 0.35) and CON. Season of enrollment (*P* < 0.001) was also associated with respiratory treatment during the first 30 days of life. Calves born in May and June were more likely to receive a treatment for respiratory diseases during the first 30 days of life than calves born in March and April (OR = 1.50; 95% CI: 0.90–2.50; *P* < 0.001). In the contrary, the odds of receiving treatment for respiratory diseases during the first 30 days of life was smaller for calves born in August and September (OR = 0.16; 95% CI: 0.07–0.33; *P* = 0.001) and in October and November (OR = 0.10; 95% CI: 0.03–0.27; *P* < 0.001) when compared to the referent group (Table 6). A Kaplan-Meier curve showing the hazard of being treated for a respiratory disease only during the first 30 days of life is presented in Figure 1B.

**Table 5:** Multivariable logistic regression evaluating the effect of the administration of a non-specific immune stimulant around transportation on disease treatment events during the rearing period (first 9 weeks of life).

**Variable**	***n*^a^**	**Disease treatment (%)**	**Odds ratio**	**95% C.I**.	***P*-value**
**Treatment**^b^					
Control	438	71 (16.2)	Referent		
BTIS	431	63 (14.4)	0.89	0.61–1.30	0.75
ATIS	436	62 (14.4)	0.89	0.61–1.30	0.71
**Season**^c^					
March–April	286	65 (22.8)	Referent		
May–June	216	51 (23.6)	1.05	0.69–1.60	0.02
August–September	485	59 (12.2)	0.70	0.44–1.10	0.71
October–November	318	21 (6.6)	0.40	0.22–0.74	0.003
Birth weight	1305	196 (15.0)	0.98	0.96–0.99	0.05
**Breed**^d^					
Jersey	568	115 (20.2)	Referent		
Jersey-cross	737	81 (11.0)	0.73	0.52–1.04	0.08
**Metaphylaxis**^e^					
No	858	161 (18.8)	Referent		
Yes	457	35 (7.8)	0.60	0.37–0.97	0.04

*The variables season of enrollment, birth weight, breed, and metaphylaxis were retained in the model*.

a *Total number of animals that had disease event (i.e., respiratory disease and/or diarrhea) in each group during the rearing period*.

b *Treatment: Animals received subcutaneous administration of 1 mL of a non-specific immune stimulant at 4 ± 1 days of life. CON = calves that receive saline before transport; BTIS = calves that received immune stimulant before transport and ATIS = immune stimulant after transport*.

c *Enrollment season: Period of the study referent to the week when first set of calves was enrolled. Calves were enrolled on a weekly basis from March to November of 2018*.

d *Breed: Jersey and Jersey-Holstein cross heifer calves were enrolled in the study*.

e *Starting in September 2018 farm management implemented a metaphylactic treatment (Zuprevo, Tildipirosin, 4 mg/kg of body weight; Merck Animal Health, Summit, NJ)*.

**Table 6:** Multivariable logistic regression evaluating the effect of the administration of a non-specific immune stimulant around transportation on the treatment of respiratory disease during the first 30 days of life.

**Variable**	***n*^a^**	**Disease treatment (%)**	**Odds ratio**	**95% C.I**.	***P*-value**
**Treatment**^b^					
Control	438	33 (7.5)	Referent		
BTIS	431	19 (4.4)	0.53	0.29–0.97	0.03
ATIS	436	32 (7.3)	0.92	0.54–1.55	0.35
**Season**^c^					
March–April	286	34 (11.9)	Referent		
May–June	216	36 (16.7)	1.50	0.90–2.50	<0.001
August–September	485	10 (2.1)	0.16	0.07–0.33	0.001
October–November	318	4 (1.3)	0.10	0.03–0.27	<0.001

*The variable season of enrollment was retained in the model*.

a *Total number of animals that had disease event (i.e., respiratory disease and/or diarrhea) in each group during the rearing period*.

b *Treatment: Animals received subcutaneous administration of 1 mL of a non-specific immune stimulant at 4 ± 1 days of life. CON = calves that receive saline before transport; BTIS = calves that received immune stimulant before transport and ATIS = immune stimulant after transport*.

c *Enrollment season: Period of the study referent to the week when first set of calves was enrolled. Calves were enrolled on a weekly basis from March to November of 2018*.

The effect of the non-specific immune stimulant around transportation and metaphylaxis on the re-treatment of Jersey and Jersey-cross calves during the rearing period is presented in Table 7. Compared with CON, there was no difference in the odds of retreatments during the rearing period for BTIS (OR = 1.53, 95% CI: 0.68–3.43; *P* = 0.20) and ATIS (OR = 0.96, 95% CI: 0.43–1.94; *P* = 0.38). Similarly, metaphylactic treatment was not associated (*P* = 0.36) with differences in retreatment during the rearing period.

**Table 7:** Logistic regression for the effects of a non-specific immune stimulant around transportation and metaphylaxis on the re-treatment of Jersey and Jersey-cross calves during the rearing period (first 9 weeks of life).

**Variable**	***n*^a^**	**Second treatment (%)**	**Odds ratio**	**95% CI**	***P*-value**
**Treatment**^b^					
Control	71	28	Referent		
BTIS	62	21	1.53	0.68–3.43	0.20
ATIS	63	30	0.96	0.43–1.94	0.38
**Metaphylaxis**^c^					
No	161	25	Referent		
Yes	35	31	0.68	0.30–1.54	0.36

a *Total number of animals that had disease event (i.e., respiratory disease and/or diarrhea) in each group during the rearing period*.

b *Treatment: Animals received subcutaneous administration of 1 mL of a non-specific immune stimulant at 4 ± 1 days of life. CON = calves that receive saline before transport (n = 438); BTIS = calves that received immune stimulant before transport (n = 431) and ATIS = immune stimulant after transport (n = 436)*.

c *Starting in September 2018 farm management implemented a metaphylactic treatment (Zuprevo, Tildipirosin, 4 mg/kg of body weight; Merck Animal Health, Summit, NJ)*.

**Figure 2:**
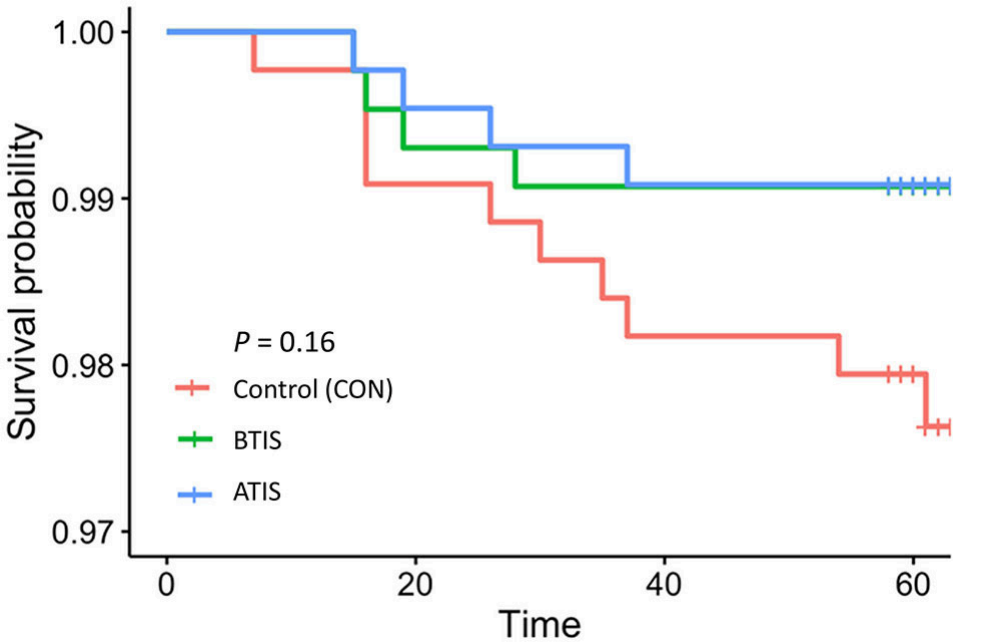
Kaplan-Meier survival curves showing the survival of calves, independently of the cause of death determined by farm personnel, during the first 9 weeks of life (time). CON = calves that receive saline before transport (*n* = 438; red line); BTIS = calves that received immune stimulant before transport (*n* = 431; green line) and ATIS = immune stimulant after transport (*n* = 436; blue line).

Mortality rates in the study population were low with 18 (1.4%) calves dying within the study period. The number of animals that died was small, so it is not surprising the differences in survivability of animals in different IS treatment groups were not statistically significant. However, the observed differences were important; the overall mortality rate during the rearing period was only 0.9% (4 deaths each) in the BTIS and ATIS groups, compared with 2.3% (10 deaths) in the control group. A marginal difference (*P* = 0.05) was observed when contrasting the mortality in both IS groups combined to the CON group (Table 4). In the multivariable logistic regression model, no statistical differences were observed even though the odds of death for BTIS and ATIS calves was 60% smaller (BTIS; OR = 0.40; 95% CI = 0.12–1.28; *P* = 0.43; ATIS; OR = 0.40; 95% CI = 0.12–1.27; *P* = 0.42) than CON calves. Similarly, a 60% lower likelihood of mortality (OR = 0.40; 95% CI = 0.16–1.01; *P* = 0.05) was observed when comparing calves from both treatment groups to calves in the CON group. Metaphylactic treatment did not influence the odds of death (OR = 0.96; 95% CI = 0.36–2.60; *P* = 0.94). Full model output is presented in Table 8 and a Kaplan-Meier curve showing the hazard of dying during the rearing period is presented in Figure 2.

**Table 8:** Multivariable logistic regression evaluating the effect of the administration of a non-specific immune stimulant around transportation on the likelihood of death during the rearing period (first 9 weeks of life).

**Variable**	***n*^a^**	**Disease treatment (%)**	**Odds ratio**	**95% C.I**.	***P*-value**
**Treatment**^b^					
Control	438	10 (2.3)	Referent		
BTIS	431	4 (0.9)	0.40	0.12–1.28	0.43
ATIS	436	4 (0.9)	0.40	0.12–1.27	0.42
**Metaphylaxis**^c^					
No	858	12 (1.4)	Referent		
Yes	457	6 (1.3)	0.96	0.36–2.60	0.94

*The variable metaphylaxis was also retained in the model*.

a *Total number of animals that had disease event (i.e., respiratory disease and/or diarrhea) in each group during the rearing period*.

b *Treatment: Animals received subcutaneous administration of 1 mL of a non-specific immune stimulant at 4 ± 1 days of life. CON = calves that receive saline before transport; BTIS = calves that received immune stimulant before transport and ATIS = immune stimulant after transport*.

c *Starting in September 2018 farm management implemented a metaphylactic treatment (Zuprevo, Tildipirosin, 4 mg/kg of body weight; Merck Animal Health, Summit, NJ)*.

## Discussion

Raising replacement heifer calves free of disease and that perform well during the rearing period results in a more productive and profitable adult dairy cow (1–4). Unfortunately, the occurrence of calf-hood diseases continues to be a challenge to dairy producers and transportation exacerbates this challenge. Dairy producers use antibiotics to treat and control diseases outbreaks and decrease mortality during the pre-weaning period (7). However, the use of antibiotics in food-producing animals has been associated with the alleged contribution of animal agriculture on the selection of antimicrobial resistance genes (18, 19). For this reason, immune stimulants that can induce early activation of the non-specific innate immune system and provide the first line of defense against microbial pathogens have emerged as an alternative to treat and prevent diseases and mortality in dairy cattle. The administration of mycobacterium cell wall fraction immune stimulant has been demonstrated to be effective on the reduction of severity, duration and mortality of induced bacterial diarrhea in dairy calves (21, 31). However, it was unknown at the start of this study whether it would be effective to improve health and performance of dairy calves transported within days of birth when they are not experimentally challenged to induce bacterial diarrhea but instead experience naturally occurring diseases.

In order to address this question, we administered the selected IS subcutaneously to newborn calves around transportation to obtain evidence that it would lead to better health and performance of dairy calves. We decided to use a 1 mL dose subcutaneously based on available information describing this dose as effective to decrease morbidity and improve weight gain in feedlot calves (20).

Considering a rearing period of 9 weeks (63 days of age), this study did not find statistically significant differences in weekly HS during the first 3 weeks, ADG, overall disease treatment rate, or mortality rate, when comparing the overall response of the administration of IS around transportation to newborn dairy calves. However, the likelihood of respiratory disease treatment during the first 30 days of life was lower for calves that received IS before transportation when compared to the calves in the control group and the percentage of calves that died during the rearing period was marginally smaller when comparing calves that received IS to calves in the control group.

The HS of all calves enrolled in our study was lower than expected and very few calves were considered to need extra attention from farm personnel based on respiratory issues. It is somewhat surprising that HS results were so low, especially following transportation. Transportation is a major cause of stress in calves (32–34), and has been associated with increased prevalence of diseases, especially respiratory diseases (14, 15). However, the differences from our results to previous reports are likely explained by the fact that calves in our study received adequate amounts of good quality colostrum, were housed individually, and were transported at a very young age. To the best of our knowledge, this is the first study to evaluate the effects of IS administered to newborn dairy calves immediately before and after transport and the first to assess HS information following IS administration and transportation. Although we expected to identify subtle biologically relevant differences in health between the different experimental groups by assessing HS during the first 3 weeks post-transport, assessing HS on a weekly basis hindered our ability to capture all the variation in HS for animals enrolled in our study. Assessing HS once per week is unlikely to capture the true incidence of diseases in a herd because the clinical signs used by the HS systems might appear and disappear in the period between two consecutive HS because of treatments given or spontaneous cure of the disease. In future studies, measurement of daily behavior and health assessments by adopting precision technologies such as activity monitoring systems and video cameras is likely to be beneficial compared to a once a week health scoring method. Lastly, caution must be applied when interpreting the respiratory HS results in our study because very few calves were deemed to need extra attention within each treatment group.

Aligned with the HS results, the differences in the ADG for calves in the different treatment groups were also not statistically significant, and were <10 g/day. Previous reports have shown that diseases during the pre-weaning period are associated with decreased growth because of decreased appetite and feed intake, and increased energy demands to support immune response (1, 35, 36). The absence of significant differences in ADG between groups in the current study agrees with other reports showing a lack of effect of immune stimulants on growth and performance of calves (37, 38), even though increased ADG was reported in feedlot calves following the administration of the same IS used in this study (20). The overall good health described in our study population contributed to the similar ADG observed in the three treatment groups. Several factors may have played a role in improving animal health in the current study including individual housing and age of the calves. Differently from other studies, in our study, calves were housed individually during the experimental period and, therefore, were less likely to experience diarrhea and respiratory problems, especially when compared to group housed calves (39). Moreover, in our study, the most stressful events (i.e., enrollment and transport) occurred within the first 4-weeks of life when passive immunity transferred from cows via colostrum provides immunologic protection to calves (40).

In our study, the disease treatment rates were lower than morbidity rates reported in the latest nationwide survey and other epidemiological studies (41, 42) and historical data from the farm where the study was conducted. A larger sample size would have been determined if a more accurate estimate of disease treatment and mortality rates were known. The lack of statistical significance for some of the analysis in our study is likely a consequence of this inadequate sample size leading to imprecise confidence intervals around the point estimates. Thus, results are discussed emphasizing estimates and the uncertainty in them as previously recommended (43).

The reduced disease treatment and mortality incidence in our study are likely related to the reduced prevalence of failure of passive transfer (96.2% of enrolled calves had >5.5 g/dL serum total solids). The transfer of passive immunity via colostrum provides neonates with immunologic protection during early life with a successful colostrum management program having 80% of the calves with serum total solids values of 5.5 g/dL or higher (28). Lastly, it is also important to keep in mind that the disease events and treatments were self-reported by farm personnel, which is a limitation of the study and could have contributed to the lower treatment rates. The authors acknowledge this limitation but we are confident that treatment assignment stayed masked for farm personnel identifying and treating sick animals, thus decreasing the risk of bias when examining and making disease treatment decisions for the study population.

In our study we observed that, in all groups, the number of calves that received treatment for respiratory disease was greater than the number of animals considered to be in need of veterinary attention based on respiratory score. In contrast, very few calves received treatment for diarrhea when compared to the number of animals considered to need veterinary attention based on fecal scores. While in an ideal scenario the disease treatment and morbidity rates would be equivalent, a discrepancy between treatment decisions by farm personnel and HS by observers using clinical score systems to identify sick calves have been described (44). Although lack of employee training on using scoring systems to make treatment decisions and discordance between scoring systems guidelines and criteria used for treatment decisions by farm personnel are probable explanations for this discrepancy (44), infrequent HS assessment and inconsistent disease recording are the likely explanation for the differences observed in our study for respiratory disease and diarrhea, respectively. Health scoring systems rely on observation of abnormal clinical signs to determine the health status of calves. However, dairy calves that exhibited abnormal clinical signs in between subsequent HS were treated by farm personnel and were unlikely to display abnormal clinical signs at the next HS assessment, accounting for the discrepancy in our dataset when comparing respiratory scores and respiratory disease treatments. These findings suggest that weekly HS likely results in under-reporting of sick calves and, therefore, should be used with caution in research studies aimed to describe respiratory disease incidence. For diarrhea treatment, farm personnel only recorded a diarrhea treatment event when administering intravenous fluids. Dairy calves with fecal score >1 received oral electrolytes in their water and our research group could not capture this treatment information in the farm management software. Thus, many more calves were considered to need veterinary attention in comparison to the number of calves that received a treatment for diarrhea.

Despite the lower disease treatment and mortality rates, important numerical differences were observed when comparing the treatment groups. It is interesting to note that calves receiving IS treatment before transportation had a significant lower likelihood of being treated for respiratory diseases during the first 30 days of life. Additionally, fewer calves that received IS administration died compared to CON. These results further support the idea that the administration of IS can induce innate immune response in calves and, consequently, decrease their susceptibility to infectious diseases (21, 31).

According to several reports, calf morbidity and mortality peaks during the first month of life with bovine respiratory disease and diarrhea as the major culprit (29, 45). The increased calf morbidity and mortality during this period is associated with reduced immunity, hence the opportunity for the use of immune stimulants. In our study, 70% of all disease treatments occurred within the first 30 days of life. The proportion of calves treated for respiratory disease within the first 30 days of life was smaller in the groups receiving IS before transportation, but no differences were observed for diarrhea. The effect of IS reducing the treatments for respiratory diseases in the current study agrees with previous work (31). We speculate that the strength of the immune response immediately after the administration of the IS and the multifactorial nature of the infectious diseases of neonatal calves played a role in this different response during the first 30 days of life. In addition, the implementation of a blanket administration of antibiotics to all animals enrolled in this study at ~35 days of life could have influenced disease progression and reduced calf morbidity and mortality, especially for the last 457 calves enrolled in the study. However, the majority of the disease treatments in the study occurred before the metaphylactic treatment. Moreover, the variable metaphylaxis was included as a covariate in our statistical models. For this reason, we did not analyze our data considering the periods pre- and post-metaphylaxis implementation separately. Although this particular management strategy introduced a potential confounding variable to the study, it also reflects the challenges inherent to performing clinical trials in commercial dairy farms.

Although the estimated difference in disease treatment and mortality were within the range described by previous studies using immune stimulants (20, 21), the rather low disease treatment and mortality rates encountered in our study may have contributed to the lack of statistically significant differences. Nonetheless, it is also possible that the administration of IS to dairy calves alters the duration of diseases events as well as the time to disease event. The authors considered this hypothesis prior to the beginning of the study, but the logistics for collecting information on disease events duration was challenging and authors decided to analyze “retreatments” as a proxy for unresolved disease cases. Unfortunately, the low disease treatment rate also resulted in a very low recurrence of disease treatments and we were unable to derive conclusions from our results. Further investigation of the effect of IS on disease events duration is warranted.

Previously published studies have shown that calves benefit from adequate transfer of passive immunity leading to fewer diseases and lower mortality, and consequently fewer antibiotic treatments (46). Although, the administration of antibiotics to newborn calves has also been associated with decreased incidence of bovine respiratory diseases and increased calf survivability (46, 47), major concerns about antibiotic resistance, antibiotic-associated diarrhea and calf-rearing costs make their continued use less favorable (48). For this reason, the results of our study provide some support for the conceptual premise that administration of IS can be another tool to improve calf health during the rearing period, especially if administered prior to transportation and periods when naturally occurring disease events are elevated. Additional studies to determine the effect of IS in multiple herds, including herds with treatment and mortality rates higher than the one reported in this manuscript are warranted to confirm the effectiveness of this intervention.

## Conclusion

The administration of IS did not significantly improve HS, ADG, and the differences in the likelihood of disease treatment within the first 9 weeks of life. However, administration of IS prior to transportation reduced the likelihood of treatment for respiratory diseases during the first 30 days of life and led to a marginal decrease in mortality during the rearing period when compared to calves that did not receive IS.

## Data Availability Statement

The raw data supporting the conclusions of this article will be made available by the authors, without undue reservation.

## Ethics Statement

The animal study was reviewed and approved by Institutional Animal Care and Use Committee (IACUC) of the University of Minnesota and Texas Tech University. Written informed consent was obtained from the owners for the participation of their animals in this study.

## Author Contributions

BO was responsible for sample collection and original manuscript draft. LC was responsible for funding acquisition, study conceptualization, and manuscript review and editing. MC, PM, DP, and AG-M were responsible for sample collection and manuscript review. VM was responsible for study conceptualization and manuscript review and editing. AM was responsible for manuscript review. AR was responsible for data analysis and manuscript review. All authors contributed to the article and approved the submitted version.

## Conflict of Interest

NovaVive Inc. employed AM during the period of this study. AM reviewed the manuscript but was not involved with any of the data analyses. The remaining authors declare that the research was conducted in the absence of any commercial or financial relationships that could be construed as a potential conflict of interest.
